# Failing stentless Bioprostheses in patients with carcinoid heart valve disease

**DOI:** 10.1186/s13019-015-0238-5

**Published:** 2015-03-27

**Authors:** Andreas Schaefer, Bjoern Sill, Jeannette Schoenebeck, Yvonne Schneeberger, Hermann Reichenspurner, Helmut Gulbins

**Affiliations:** Department of Cardiovascular Surgery, University Heart Center Hamburg, Martinistraße 52 20246 Hamburg, Germany

**Keywords:** Carcinoid heart valve disease, Hedinger’s syndrome, Pulmonal valve, Pulmonal valve replacement, Tricuspid valve, Stentless bioprosthesis, Bioprosthesis

## Abstract

**Background:**

Carcinoid tumor with consecutive endocardial fibroelastosis of the right heart, known as carcinoid heart valve disease (CHVD) or Hedinger’s syndrome, is accompanied by combined right-sided valvular dysfunction with regurgitation and stenosis of the affected valves. Cardiac surgery with replacement of the tricuspid and/or pulmonary valve is an established therapeutic option for patients with Hedinger’s syndrome. Little is known about the long term outcome and the choice of prosthesis for the pulmonal position is still a matter of debate.

**Methods:**

The authors report three cases of pulmonary valve replacement with stentless bioprostheses (Medtronic Freestyle®, Medtronic PLC, Minneapolis, MN, USA) due to severe pulmonary valve degeneration in consequence of Hedinger’s syndrome.

**Results:**

All patients presented with re-stenosis of the pulmonal valve conduit at the height of the anastomoses in a premature fashion. Due to the increased risk for repeat surgical valve replacement, two patients were treated by transcatheter heart valves.

**Conclusion:**

We do not recommend the replacement of the pulmonary valve with stentless bioprostheses in patients with CHVD. These valves presented with an extreme premature degeneration and consecutive re-stenosis and heart failure.

## Background

The carcinoid syndrome with consecutive endocardial fibroelastosis of the right heart was first described by Christoph Hedinger as a “Metastatizing Carcinoid with tricuspid valve degeneration and stenosis of the pulmonal valve-(a new syndrome)” in 1954 [1]. Carcinoid tumors have an incidence of 1.2 to 2.1 per 100,000 persons and are characterized by flush, diarrhea and acute asthma attacks [2,3]. The tumor releases vasoactive substances such as serotonin in the portal circulation, which can be deactivated through the liver. When liver metastases appear serotonin is secreted in the systemic circulation and carcinoid heart and valve disease (CHVD) known as Hedinger’s syndrome occur [4]. Replacement of the tricuspid and pulmonary valve is a well established and a feasible therapeutic option for these patients [5]. Nevertheless little is known in terms of long term outcome and the choice of the valve prosthesis is still a matter of debate. Here we report three cases of pulmonary valve replacement with the Medtronic Freestyle® (Medtronic PLC, Minneapolis, MN, USA) stentless bioprosthesis in patients with CHVD. All three of these patients presented with pulmonary valve degeneration and were also treated with concomitant tricuspid valve replacement due to severe tricuspid valve insufficiency.

## Methods

### Case 1

**Figure 1:**
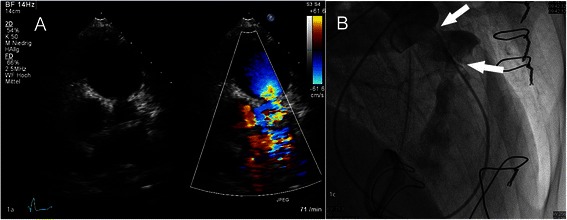
**Transthoracic echocardiography and angiography of patient no. 1. A**: Transthoracic echocardiography of the degenerated pulmonal valve prosthesis in doppler color flow with signs of stenosis. **B**: Angiography with stenosis of the proximal and distal anastomoses **(see markers)** of the bioprosthesis.

A 34-year-old male patient was transferred to our institution in June 2011 with cyanosis and new diagnosed severe pulmonary valve stenosis (PS) and a severe tricuspid valve insufficiency (TI). The patient suffered from asthma, diarrhea and flush for two years. Transthoracic echocardiography (TTE) revealed a normal left-ventricular (LV) function and a mildly reduced right-ventricular (RV) function. The transvalvular gradient of the pulmonary valve was elevated up to 59/19 mmHg (peak/mean) with concomitant severe insufficiency due to shortened and thickened leaflets. The tricuspid valve also showed severe insufficiency. The systolic pulmonary artery pressure (sPAP) was 43 mmHg. Pulmonary embolism could be ruled out by right heart catheter. Abdominal CT presented a tumor in the midgut and metastases of the liver. Biopsy specimen of the liver confirmed the clinical diagnosis of a CHVD. In August 2011 the patient underwent cardiac surgery. The pulmonary valve was replaced with a Medtronic Freestyle® (25 mm) and the tricuspid valve with an Edwards Perimount® (31 mm) pericardial valve. Additionally a patch plasty of the pulmonal artery bifurcation was performed to avoid distal stenosis of the pulmonary conduit. Detailed procedural data are summarized in Table 1. At time of discharge the TTE presented trace TI and trace PI with a transprothetic gradient of 14/5 mmHg (peak/mean). Sandostatin therapy was induced. In January 2012 resection of the midgut tumor and partial resection of liver metastasis was done. The patient was admitted again to our centre in June 2012 for transarterial chemoembolization (TACE) of the residual liver metastasis. At time of admission the patient presented with cardiac decompensation due to a severe re-stenosis of the pulmonary valve conduit with a pressure gradient of 90/48 mmHg. The tricuspid valve bioprosthesis presented a regular function. After an uncomplicated TACE procedure, a balloon-valvuloplasty of the pulmonary valve conduit was performed with a postprocedural decrease of the peak pressure gradient to 30 mmHg. In June 2013 the patient was referred to our centre with relapse of right heart failure. The TTE now revealed a transprosthetic peak pressure gradient of the pulmonary valve conduit of 80 mmHg (Figure 1A). Angiography of the right heart presented a regular function of the valve itself, but stenosis of the proximal conduit-anastomosis with a diameter of 12 mm and stenosis of the distal conduit-to-pulmonal artery anastomosis with a diameter of 11.8 mm (Figure 1B). A successful balloon dilation of the distal stenosis was performed followed by a staged percutaneous, transvenous stent implantation into the pulmonary artery and subsequent implantation of a balloon-expandable transcatheter heart valve in September 2013. At 6 month follow up echo presented a regular function of the pulmonal valve.

**Table 1:** Procedural data*

	Patient 1	Patient 2	Patient 3
Pulmonal valve replacement	Medtronic Freestyle 25 mm	Medtronic Freestyle 25 mm	Medtronic Freestyle 25 mm
Tricuspid valve replacement	Edwards Perimount 31 mm	Edwards Perimount 29 mm	Edwards Perimount 25 mm
Concomitant procedure	Patch plasty of the pulmonal artery bifurcation	Patch plasty of the pulmonal artery bifurcation	Patch plasty of the pulmonal artery bifurcation
Operation time (min)	240	180	300
Cross clamp time (min)	102	79	118
CPB time (min)	161	124	195

CPB- cardiopulmonal bypass, Min- minutes.

*All surgical procedures were performed with support of ECC via bicaval and aortic canulation.

### Case 2

**Figure 2:**
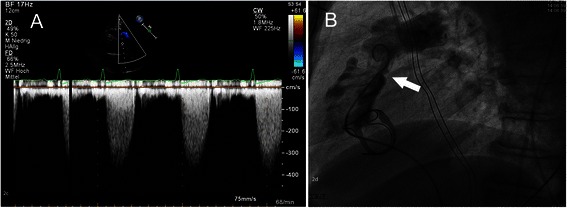
**Transthoracic echocardiography and angiography of patient no. 2. A**: Transthoracic echocardiography of the degenerated pulmonal valve prosthesis in continuous wave doppler with signs of stenosis. **B**: Angiography of the bioprosthesis with stenosis of the proximal anastomosis **(see marker)**.

A 58-year-old female patient was transferred to our institution as emergency admission in January 2011 with congestive heart failure (New York Heart Association [NYHA] Class II) and peripheral edema. Besides the patient suffered from breast cancer and was treated with two complete cycles of adjuvant chemotherapy. The TTE revealed a mildly reduced LV and RV function with a severe tricuspid valve insufficiency and a moderate stenosis. The pulmonary valve showed a moderate insufficiency with a pressure gradient of 14/5 mmHg. In May 2011 carcinoma tumor of the midgut and multiple liver metastases could be verified by MRT and liver biopsy. In June 2011 colon-, ovar- and cholecystektomy were performed without complications. Sandostatin therapy was induced. The patient was transferred as emergency admission to our institution again in September 2011 due to relapsed cardiac decompensation with ascites and severe dyspnea (NYHA III-IV). The pulmonary valve now presented with a pressure gradient of 18/9 mmHg. In February 2012 pulmonary valve replacement with a Medtronic Freestyle® (25 mm) and tricuspid valve replacement with an Edwards Perimount® (29 mm) was performed. Additionally a patch plasty of the pulmonal artery bifurcation was done. At time of discharge tricuspid and pulmonary bioprostheses presented with a trace insufficiency. In April 2013 the patient presented again with heart failure symptoms. The TTE presented a regular function of the tricuspid bioprosthesis. The conduit of the pulmonary valve was severely stenotic with a consecutive pressure gradient of 57/34 mmHg (Figure 2A). Right heart catheter exposed a stenosis of the proximal conduit-anastomosis with a diameter of 11 mm. The valve and the distal conduit- anastomosis showed no significant stenosis (Figure 2B). In September 2013 transvenous interventional valve-in-valve procedure was performed as described in case 1. At 6 month follow up newly occurred metastases of the ovar were found. The pulmonal valve showed a regular function.

### Case 3

**Figure 3:**
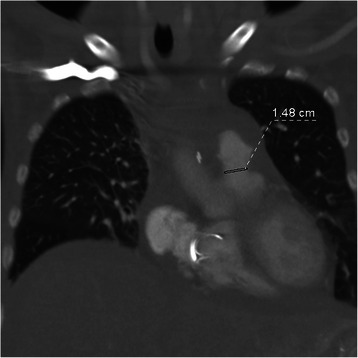
**Computed tomography of the thorax with stenosis of the distal anastomosis of the pulmonary valve conduit with a diameter of 14.8 mm.**

A 51-year-old female patient was transferred to our institution in May 2011 with a known carcinoid tumor, progressive dyspnea (NYHA III) and progressive peripheral edema for the last two years. The neuroendocrine tumor of the ileum was first diagnosed in 2008. Midgut resection was performed in 2008 and liver metastases were verified by CT. Sandostatin therapy was induced postoperatively. Significant coronary artery disease requiring intervention was ruled out by angiography. The TTE revealed a severe tricuspid valve insufficiency and a pressure gradient of the pulmonary valve of 13/8 mmHg. Diagnosis of Hedinger’s syndrome was confirmed and tricuspid valve replacement was performed in June 2011 with a Medtronic Hancock® (27 mm). At time of discharge the tricuspid bioprosthesis presented a regular function and clinical condition showed significant improvement with dyspnea NYHA I and no peripheral edema. In September 2011 the patient was transferred again to our institution due to recurrence of dyspnea and peripheral edema. The TTE presented a regular function of the tricuspid valve bioprosthesis but a severe insufficiency and a moderate stenosis of the pulmonary valve with a pressure gradient of 23/12 mmHg. After recompensation the patient could be discharged. In June 2012 the patient presented again with right heart failure. Leading clinical symptoms were dyspnea, peripheral edema and additionally severe ascites. The echo now revealed a moderate insufficiency of the tricuspid valve bioprosthesis and the pulmonary valve was severely insufficient and moderately stenotic. The right heart function was reduced (Tricuspid annular plane systolic excursion [TAPSE] of 8 mm). Implantation of a Medtronic Melody® valve through right heart catheter was terminated due to a rather small annulus diameter of the pulmonary valve (23 mm). In December 2012 re-tricuspid valve replacement and pulmonary valve replacement were performed. Intraoperatively no early degeneration of the tricuspid valve was seen. The insufficiency was a result of a compromised leaflet due to a papillary muscle. The pulmonary valve was replaced with a Medtronic Freestyle® 25 mm. Additionally a patch plasty of the pulmonary artery bifurcation was done to avoid stenosis of the distal anastomosis. The postoperative echo showed a regular function of both prostheses. Unfortunately the patient died in January 2013 due to a complication during TACE procedure. Computed tomography 10 days prior to the TACE procedure presented a stenotic distal anastomosis of the pulmonary valve conduit with a diameter of 14.8 mm (Figure 3).

### Consent

Written informed consent was obtained from the patient’s guardian/parent/next of kin for the publication of this report and any accompanying images.

## Results

Here we reported three cases of pulmonary valve replacement with stentless bioprostheses (Medtronic Freestyle®) in patients suffering from CHVD. All patients presented with re-stenosis at the height of the anastomoses of the valve conduit in a premature fashion. Hence, the re-stenosis, although clinically remarkable, was not a problem of the valve itself. Due to the increased risk for repeat surgical valve replacement, two patients were treated by percutaneous stent implantation into the pulmonary artery, followed by the implantation of a balloon-expandable transcatheter heart valve.

## Discussion

CHVD almost exclusively affects right-sided heart valves, since circulating serotonin is metabolized by pulmonary endothelial cells, thus sparing left-sided valves. Although the pathophysiology of CHVD is not fully understood, certain distinct features of this entity have been well recognized. The deposition of carcinoid fibrotic tissue usually begins on the downstream aspect - that is, the ventricular aspect of the tricuspid. valve and the arterial aspect of the pulmonary valve -leading to a thickening of valvular tissue with the subsequent restriction of leaflet motion. The final stage is a combined valvular dysfunction with regurgitation and stenosis of the affected valves [6]. Surgical valve replacement is usually necessary in advanced stages of the disease. Whether biological valve substitutes are subject to a continuous degenerative process is not entirely clear at present due to the rarity of the disease and inconclusive findings in the current literature [7].

Despite that, replacement of degenerated heart valves due to Hedinger’s syndrome is an established therapeutic option [5]. Especially replacement of the tricuspid valve with a stented bioprosthesis seems to offer an acceptable lifespan with no early valve deterioration and is supported by the above mentioned clinical and surgical cases [7]. In contrast the appropriate valve type for pulmonary valve replacement is still a matter of debate. Voigt et. al suggested a stentless bioprosthesis for the pulmonary valve position to avoid anticoagulation prior to hepatic de-arterialization [8]. Contrary to that Mokhlesa and colleagues showed that mechanical valve prostheses are not accompanied by valve related complications such as bleeding or thrombosis [9].

In the presented cases we performed pulmonary valve replacement with stentless bioprostheses and a concomitant patch plasty, which is compellent at the time of replacement. The patch plasty was used to increase the RVOT to pulmonary valve annulus continuum. The idea was to implant valves greater in diameter and avoid at least a re-stenosis involving the pulmonary artery. This principle could not be proved in our case series since re-stenosis of the anastomoses occurred in all our patients regardless of the performed patch plasty. In two of the described cases our patients required interventional treatment due to stenotic lesions involving the anastomoses of the valve replacement. Extreme premature degeneration within 1.5-2 years after implantation should be avoided due to consecutive recurrence of heart failure and mortality, respectively.

The two patients who were treated by percutaneous stent implantation into the pulmonary artery, followed by the implantation of a balloon-expandable transcatheter heart valve presented a favorable acute outcome, with adequate valve function and no paravalvular leakage as documented by TTE, although the transvalvular gradients were elevated in both cases. The patients had an uneventful postoperative course and were discharged home in timely fashion. Both patients showed a normal ejection fraction of the right ventricle in the 6 month follow-up.

## Conlusions

We can not recommend the replacement of the pulmonary valve with stentless bioprostheses in patients with CHVD. Maybe stented bioprostheses or mechanical prostheses with no anastomoses with foreign material have the potential to avoid re-stenosis. The short durability of stentless bioprostheses and therefore the higher incidence of redo-surgery or intervention, which was shown in our cases, led us to the strong conviction to not utilize stentless bioprostheses in pulmonal position in patients with CHVD, prospectively.

## Abbreviations

CHVD: Carcinoid heart valve disease
LV: Left ventricle/ventricular
NYHA: New York heart association
PS: Pulmonary valve stenosis
RV: Right ventricle/ventricular
sPAP: systolic pulmonary artery pressure
TACE: Transarterial chemoembolization
TAPSE: Tricuspid annular plane systolic excursion
TI: Tricuspid valve insufficiency
TTE: Transthoracic echocardiography

## References

[1] Hedinger C, Gloor R (1954). Metastasierende Dünndarmkarzinoide, Tricuspidalklappenveränderungen und Pulmonalstenose- ein neues Syndrom. Schweizerische Medizinische Wochenschrift.

[2] Modlin IM, Sandor A (1997). An analysis of 8305 cases of carcinoid tumors. Cancer.

[3] Melmon KL, Sjoerdsma A, Mason DT (1965). Distinctive clinical and therapeutic aspects of the syndrome associated with bronchial carcinoid tumors. Am J Med.

[4] Bernheim AM, Connolly HM, Pellikka PA (2007). Carcinoid heart disease in patients without hepatic metastases. Am J Cardiol.

[5] Connolly HM, Nishimura RA, Smith HC, Pellikka PA, Mullany CJ, Kvols LK (1995). Outcome of cardiac surgery for carcinoid heart disease. J Am Coll Cardiol.

[6] Roberts WC, Sjoerdsma A (1964). The cardiac disease associated with the carcinoid syndrome (carcinoid heart disease). Am J Med.

[7] Mabvuure N, Cumberworth A, Hindocha S (2012). In patients with carcinoid syndrome undergoing valve replacement: will a biological valve have acceptable durability?. Interact Cardiovasc Thorac Surg.

[8] Voigt PG, Braun J, Teng OY, Dion R (2005). Double Bioprosthetic valve replacement in right-sided carcinoid heart disease. Ann Thorac Surg.

[9] Mokhlesa P, van Herwerden LA, van Domburga RT, Roos-Hesselinka JW (2012). Carcinoid heart disease: outcomes after surgical valve replacement. Eur J Cardiothorac Surg.

